# Liver regeneration therapy through the hepatic artery-infusion of cultured bone marrow cells in a canine liver fibrosis model

**DOI:** 10.1371/journal.pone.0210588

**Published:** 2019-01-23

**Authors:** Tatsuro Nishimura, Taro Takami, Ryo Sasaki, Yuki Aibe, Takashi Matsuda, Koichi Fujisawa, Toshihiko Matsumoto, Naoki Yamamoto, Kenji Tani, Yasuho Taura, Isao Sakaida

**Affiliations:** 1 Department of Gastroenterology & Hepatology, Yamaguchi University Graduate School of Medicine, Ube, Yamaguchi, Japan; 2 Center for Regenerative and Cell Therapy, Yamaguchi University Organization for Research Initiatives, Ube, Yamaguchi, Japan; 3 Center for Reparative Medicine, Yamaguchi University Graduate School of Medicine, Ube, Yamaguchi, Japan; 4 Department of Oncology and Laboratory Medicine, Yamaguchi University Graduate School of Medicine, Ube, Yamaguchi, Japan; 5 Yamaguchi University Health Administration Center, Yamaguchi, Yamaguchi, Japan; 6 Department of Veterinary Surgery, Joint Faculty of Veterinary Medicine, Yamaguchi, Yamaguchi, Japan; University of Navarra School of Medicine and Center for Applied Medical Research (CIMA), SPAIN

## Abstract

**Background:**

We previously reported regenerative therapies for decompensated cirrhosis based on peripheral venous drip infusion using non-cultured whole bone marrow (BM) cells, or the less invasive cultured BM-derived mesenchymal stem cells (BMSCs). Here, we assessed the efficacy and safety of hepatic arterial infusion using cultured autologous BMSCs, comparing it with peripheral infusion, using our established canine liver fibrosis model.

**Methods:**

Canine BM cells were harvested and cultured, and the resultant BMSCs were returned to carbon tetrachloride (CCl_4_)-induced liver cirrhosis model canines via either a peripheral vein (Vein group) or hepatic artery (Artery group). A variety of assays were performed before and 4, 8, and 12 weeks after BMSC infusion, and liver fibrosis and indocyanine green (ICG) half-life (t_1/2_) were compared to those in a control group that received CCl_4_ but not BMSCs. The safety of this approach was evaluated by contrast-enhanced computed tomography (CT) and serial blood examinations after infusion.

**Results:**

Four weeks after infusing BMSCs, a significant improvement was observed in the Vein group (n = 8) compared to outcome in the Control group (n = 10), along with a decrease in ICG t_1/2_. In the Artery group (n = 4), ICG t_1/2_ was significantly shorter than that in the Vein group at 8 weeks (Δt_1/2_: −3.8 ± 1.7 min vs. +0.4 ± 2.4 min; p < 0.01) and 12 weeks (Δt_1/2_: −4.2 ± 1.7 min vs. +0.4 ± 2.7 min; p < 0.01) after BMSC administration. Post-infusion contrast-enhanced CT showed no liver infarction, and blood tests showed no elevations in either serum lactate dehydrogenase concentrations or hypercoagulability.

**Conclusions:**

We confirmed the efficacy and safety of the hepatic arterial infusion of cultured autologous BMSCs using a canine model, thereby providing non-clinical proof-of-concept.

## Introduction

Liver cirrhosis represents the advanced stage of liver fibrosis, presenting as regenerative nodules surrounded by fibrotic bands that develop in response to chronic liver injury [1–3]. Currently, the most effective therapy for advanced cirrhosis is liver transplantation. However, the procedure has several limitations including the lack of donors, surgical complications, immunological suppression, and high medical costs [4]. Therefore, novel alternative therapies for decompensated liver cirrhosis are urgently needed.

In recent years, several studies using animal models of liver diseases have demonstrated that bone marrow (BM) cell transplantation might accelerate liver regeneration and reduce hepatic fibrosis [5–8].

Accordingly, we developed autologous BM cell infusion (ABM*i*) therapy for liver cirrhosis and reported its efficacy and safety for liver cirrhosis [9,10]. However, since ABM*i* therapy requires BM aspiration under general anesthesia, some patients are excluded due to poor liver or cardiopulmonary functions. In an effort to expand the applicability of ABM*i* therapy, we developed a less invasive method for liver regeneration therapy using autologous BM-derived mesenchymal stem cells (BMSCs) cultured from small amounts of BM fluid aspirated under local anesthesia. However, before human clinical trials can be considered, the safety and efficacy of cultured autologous BMSC infusion must be confirmed in medium-to-large animals. To this end, we previously established a useful canine liver fibrosis model generated through the repeated administration of carbon tetrachloride (CCl_4_) through a catheter for 10 weeks. Using this model, we found that peripheral venous infusion using cultured autologous BMSCs could improve liver fibrosis without adverse effects, suggesting that this could represent a less invasive therapeutic option [11].

Here, to develop liver regeneration therapy with enhanced therapeutic effects, we evaluated the efficacy and safety of the hepatic arterial infusion of cultured autologous BMSCs, comparing it with peripheral infusion, using our established canine model of liver fibrosis.

## Materials and methods

### Animals and ethics

Twenty-two beagles (1–2 years of age; 10 males and 12 females) were used in this study. These animals included six canines in the control group and four in the peripheral venous infusion group, from which 0- and 4-week data were used from our previous study [11]. These canines were housed in our animal facility and treated in accordance with the university’s Animal Care Guidelines. In particular, they were housed in individual metal cages (100 cm in width and 120 cm in height) and given standard solid food (Oriental Yeast Co., Tokyo, Japan), according to dietary recommendations that were based on body weight, age, and activity level of each canine. They had free access to water at all times and were housed in a climate-controlled building at 22–23 °C, with a 16-h light/8-h dark cycle. They were taken for walks daily in the evening (15:00 to 17:00) and were inspected at least three times per day to monitor their health and well-being. Facility staff members including our group checked several health conditions as follows: appetite, thirst, defecation, urination, behavior and lethargy, etc. After surgery, we also checked these health conditions at least three times per day. This study was approved by the Institutional Animal Care and Use Committee of Yamaguchi University, “Ube” area. (Approval number: 21–033).

### Catheter implantation

All catheter implantations were performed as previously described [11]. Briefly, this was performed under general anesthesia for all canines. The stomach was directly punctured with an 18-gauge Teflon IV catheter (6-French P-U catheter; Toray Medical Co. Ltd., Tokyo, Japan). An infusion port (P-U Celsite port; Toray) was then placed in a subcutaneous pocket created on the back. Carbon tetrachloride (CCl_4_, Wako, Osaka, Japan) was administered from the port, which essentially represented oral administration. Intramuscular buprenorphine at 10 μg/kg body weight (BW) and subcutaneous cefovecin (Convenia; Zoetis Japan, Tokyo, Japan) at 8 mg/kg BW were given at the end of the procedure for post-operative analgesia and to prevent infections, respectively. As stated, we checked several health conditions after surgery at least three times per day.

### Aspiration of bone marrow fluid, culture, and infusion of BMSCs

After catheter implantation, a 16-gauge biopsy needle (Angiotech, Gainesville, FL) was used to puncture the proximal humerus of each canine and BM was aspirated using a 5-mL syringe filled with 1 mL of heparin. To culture BMSCs, the aspirated BM fluid was seeded in T-75 flasks (Life Technologies Corp., Grand Island, NY) and cultured with 10% fetal bovine serum (Life Technologies) and 100 μg/mL gentamicin (Life Technologies) in Dulbecco’s modified Eagle Medium (DMEM, Life Technologies) in a 5% CO_2_ incubator at 37 °C. After 2 days of incubation, non-adherent cells were removed during medium replacement. The culture medium was changed every 2 days for 2–3 weeks. Expanded BMSCs were detached using cell detachment solution and then collected.

### Experimental model (induction of liver fibrosis)

**Fig 1:**
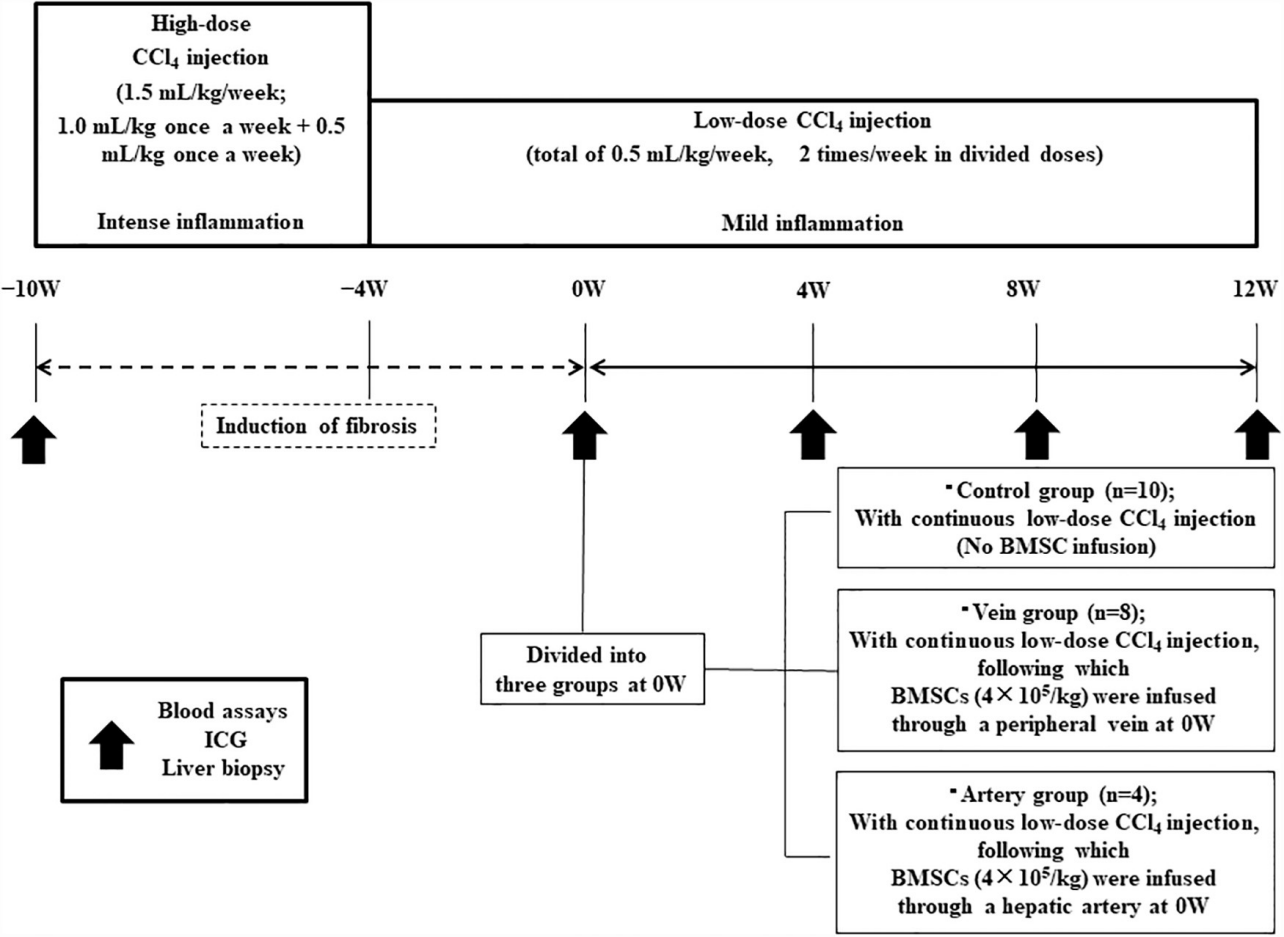
Experimental protocol for canine model of liver fibrosis and treatment. The canines were administered repeated carbon tetrachloride (CCl_4_) injections through the implanted catheter for 10 weeks (high-dose period: 1.0 mL/kg body weight once per week and 0.5 mL/kg body weight once per week for 6 weeks; low-dose period: 0.25 mL/kg body weight twice per week for 4 weeks). CCl_4_ was injected on the first and fourth days of each week. At 0 weeks (0W; i.e. after 10 weeks of CCl_4_ injections), the canines were divided into three groups. In the control group, low-dose CCl_4_ injection was continued for a further 12 weeks without bone marrow-derived mesenchymal stem cell (BMSC) infusion. In the Vein group, cultured autologous canine BMSCs (4 × 10^5^/kg) were infused through a peripheral vein, and low-dose CCl_4_ injections were continued for 12 more weeks. In the Artery group, cultured autologous BMSCs (4 × 10^5^/kg) were infused through a hepatic artery using angiography, and low-dose CCl_4_ injections were continued for 12 more weeks. In all three groups, blood assays, indocyanine green (ICG) testing, and liver biopsy based on ultrasonography were performed at −10W, 0W, 4W, 8W, and 12W, as indicated by the arrows.

CCl_4_ was diluted 1:1 in corn oil before injecting it intra-gastrically via the catheter. Injections ware repeated for 10 weeks using the implanted catheter (high-dose period: 1.0 mL/kg BW once per week and 0.5 mL/kg BW once per week for 6 weeks; low-dose period: 0.25 mL/kg BW twice per week for 4 weeks) to induce liver fibrosis (Fig 1). After the infusion of BMSCs, low-dose CCl_4_ was further administrated for 12 weeks. CCl_4_ injections were performed on the first and fourth days of each week.

### Experimental groups

After 10 weeks of CCl_4_ injections, the 22 canines were divided into three groups including the Control, Vein, and Artery groups. This point was defined as “0W”. Subsequently, the 10 animals in the Control group received low-dose CCl_4_ injection for a further 12 weeks. The eight canines in the Vein group were infused with cultured autologous BMSCs (4 × 10^5^/kg) through a peripheral vein and low-dose CCl_4_ injection was continued for 12 more weeks. The four canines in the Artery group were infused with cultured autologous BMSCs (4 × 10^5^/kg) through a hepatic artery using angiography, and low-dose CCl_4_ injection was continued for 12 more weeks.

Serial examinations including blood tests, ultrasonography-guided liver biopsies, and indocyanine green (ICG) tests were performed before and 10, 14, 18, and 22 weeks after commencing CCl_4_ injection (i.e. at −10W, 0W, 4W, 8W and 12W; Fig 1). Furthermore, abdominothoracic contrast-enhanced computed tomography (CE-CT) was performed 30 min after BMSC-infusion to evaluate the presence of liver infarction and portosystemic shunting (PSS).

### Safety evaluations with an increased BMSC dose

BMSCs were increased to 1.2 × 10^6^ cells/kg and infused through a hepatic artery into the canine model of liver fibrosis to test the effects of a higher dose (n = 1). The general condition and vital signs were monitored before and after BMSC infusion. Blood parameters were measured before BMSC infusion and at 1, 3, 5, and 7 days after infusion. Seven days after BMSC infusion, after collecting blood samples, we conducted a liver biopsy to evaluate the presence of liver infarction based on histological analysis.

### Laboratory tests

The blood samples collected at the stipulated time points were used to assess serum albumin, alanine aminotransferase, aspartate aminotransferase, bilirubin, prothrombin time PT, anti-thrombin3 (AT3) activity, and fibrin degradation products.

### ICG tests to assess liver functions

A 22-G indwelling venous cannula was inserted into the internal jugular vein and blood was collected. Following the collection of blood for the 0 time-point sample, 0.1 mg/kg of ICG (Diagnogreen Inj.; Daiichi Sankyo Co. Ltd., Tokyo, Japan) was administered via the peripheral vein and blood was collected 5, 10, 15, 20, and 30 min later from the maintained venous route. Blood samples were then centrifuged and sera were harvested from blood samples and analyzed for ICG content using an ICG Meter (Fuchu Giken, Inc., Tokyo, Japan) The difference in the ICG half-life (t_1/2_), or ΔICG (min), was calculated using the formula, ΔICG (min) = ICG t_1/2_ (min) at 4W, 8W, or 12W –ICG t_1/2_ (min) at 0W.

### CE-CT imaging

CE-CT examinations were performed using an eight-detector-row CT system (ECLOS 8; Hitachi Medical Corp., Tokyo, Japan). All canines were first placed in ventral recumbency and all CT scans were performed during apnea under anesthesia. Canines were administered 2 mL/kg iopamidol with an iodine concentration of 370 mg/mL (Oiparomin 370; Fuji Pharmaceutical Co., Toyama, Japan) as intravenous contrast medium.

### Liver biopsy

After the intramuscular injection of 20 μg/kg medetomidine (Dorbene; Kyoritsu Seiyaku Corp., Tokyo, Japan), the canines were placed in the left lateral decubitus position. The skin over the biopsy site was shaved and then disinfected with povidone-iodine. The local anesthetic (0.5% lidocaine; Pfizer, Tokyo, Japan) was injected into a small area of the skin and into tissues over part of the liver, and ultrasonography-guided liver biopsies were collected using a 16-gauge biopsy needle (Aragon Medical Devices, Plano, TX). After removing the biopsy needle, the puncture site was manually compressed and atipamezole hydrochloride was injected intramuscularly.

Some samples were fixed in 4% paraformaldehyde overnight and used for the evaluation of liver fibrosis by Sirius-red staining, whereas others were stored at −80 °C for real-time quantitative polymerase chain reaction (RT-PCR). These experiments including ultrasonography-guided liver biopsies were performed over time. We analyzed gene expression related to liver fibrosis by RT-PCR using ultrasonography-guided biopsied specimens; therefore, we have been taking care of these canines after this study, and we did not sacrifice them.

### Sirius-red staining

Paraffin-embedded liver samples were sectioned (3-μm) and stained with Sirius-red staining. For this, paraffin sections were de-waxed, hydrated, and stained with Sirius-red solution (1.3% aqueous picric acid/0.1% Direct Red 80) for 15–20 min to detect liver fibrosis. Samples were then washed with 0.5% acetic acid for 1 min and dehydrated using 100% ethanol for 5 min. Samples were cleared in xylene for 10 min and then mounted in a resinous medium.

### Histomorphometry

Histomorphometry was performed using an imaging system coupled to a fluorescence microscope (Biorevo BZ9000; Keyence, Osaka, Japan). The fibrotic area was calculated as the percent Sirius red-stained area relative to the total sample using a BZ Analyzer II (Keyence). Vessels stained with Sirius red were excluded from the calculation. We defined the percent fibrotic area as the fibrosis level. The difference in the fibrotic area, or Δfibrosis level (%), was calculated using the formula Δfibrosis level (%) = fibrosis level (%) at 4W, 8W, or 12W − fibrosis level (%) at 0W.

### Angiography and intra-arterial BMSC injection

**Fig 2:**
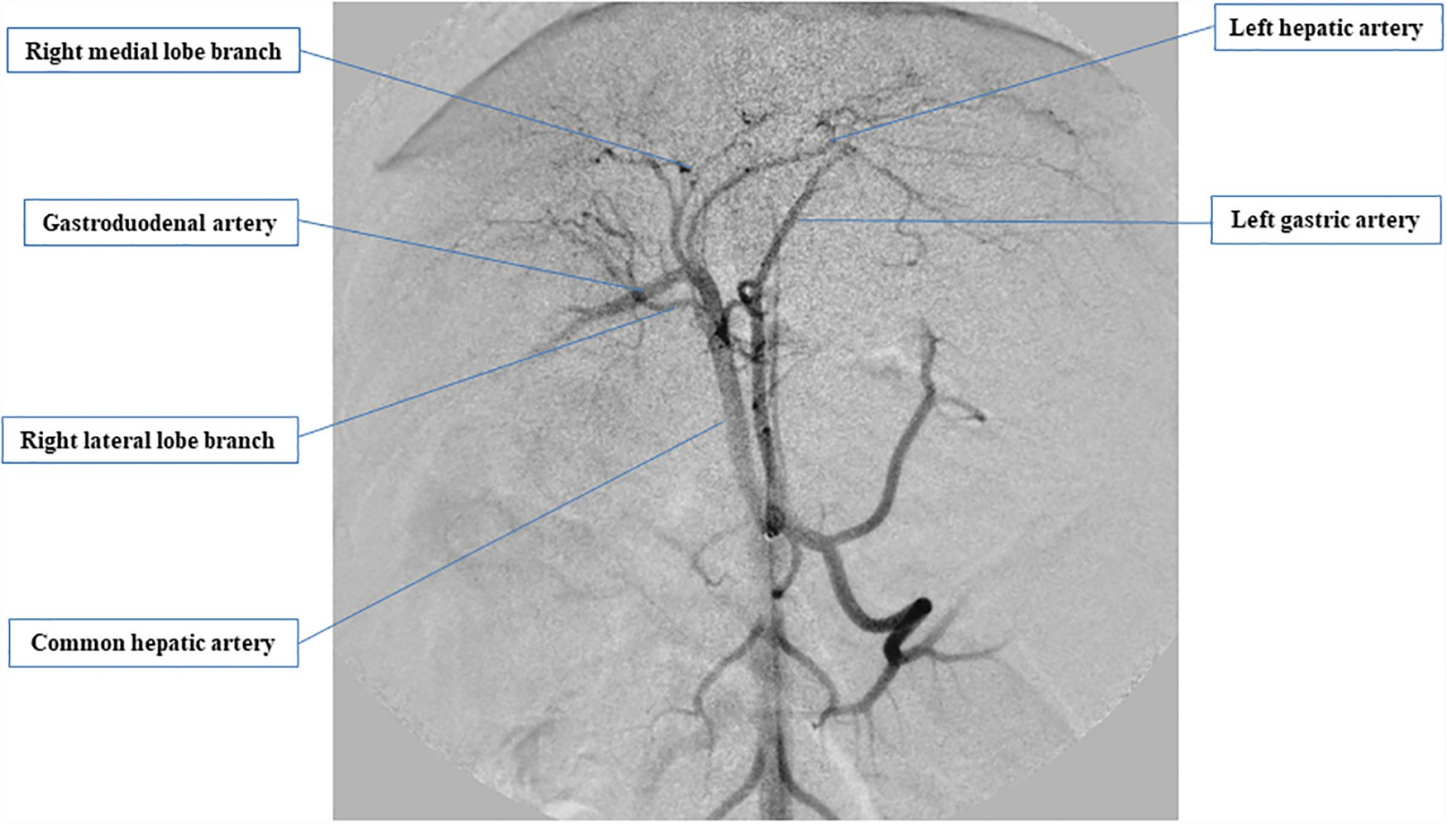
Ventral-dorsal digital subtraction images of a canine. Hepatic arteriogram was performed after the injection of contrast medium through a 4-French catheter placed in the common hepatic artery. We inserted a catheter in the left hepatic artery, right medial lobe branch, and right lateral lobe branch and injected bone marrow-derived mesenchymal stem cells (BMSCs) via each of these arteries at doses of 2 × 10^5^ cell/kg, 1 × 10^5^ cell/kg, and 1 × 10^5^ cell/kg, respectively.

Anesthesia was induced via the slow intravenous administration of propofol (1% intravenous propofol, 7 mg/kg; Maruishi Pharmaceutical Co, Osaka, Japan) and maintained with isoflurane (isoflurane, 1.4–2.5%; Dainippon Sumitomo Pharma Co, Tokyo, Japan) and oxygen. All canines were administered an antibiotic (cefazolin sodium, 25 mg/kg intravenously) and analgesic (buprenorphine, 20 mg/kg intramuscularly) after induction. The right femoral artery was punctured with a 20-G needle and cannulated with a 4-French (Fr) introducer sheath (Radifocus introducer sheath; Terumo Co, Tokyo, Japan). A guidewire (SURF, diameter: 0.89 mm, angled, 80 cm; Piolax Medical Devices Inc., Yokohama, Japan) and catheter (PA catheter, 4 Fr, 40 cm; Terumo Clinical Supply Co, Gifu, Japan) were inserted into the aorta and celiac artery with fluoroscopic guidance (ARCADIS Varic; Siemens Healthcare Japan, Tokyo, Japan). A microguidewire (Radifocus guidewire, diameter: 0.41 mm, angled, 100 cm; Terumo Clinical Supply Co.) was advanced into the common hepatic artery through the catheter. Then, a 2.1-Fr microcatheter (Sniper 2, 80 cm; Terumo Clinical Supply Co, Gifu, Japan) was placed in the common hepatic artery prior to injection of the contrast agent (Optiray 350; Covidien Co, Tokyo, Japan) under digital subtraction angiography (Fig 2).

Next, we inserted a catheter into the left hepatic artery, right medial lobe branch, and right lateral lobe branch and administered BMSCs via each of these arteries at doses of 2 × 10^5^ cells/kg, 1 × 10^5^ cells/kg, and 1 × 10^5^ cells/kg, respectively. After removing the sheath, the puncture site was manually compressed. Cefazolin sodium (25 mg/kg, intravenously) and buprenorphine (20 mg/kg, intramuscularly) were administered after treatment.

### RT-PCR analysis

Total RNA extraction was performed on liver biopsy specimens using an RNeasy Mini kit (Qiagen GmbH, Hilden, Germany). For cDNA synthesis, Taqman reverse transcription reagents were used as described in the manufacturer’s manual (Roche Diagnostics, Indianapolis, IN). Variations in gene expression were analyzed using a Step One Plus real-time PCR system (Life Technologies) with SYBR green. Relative quantification of gene expression was performed using *ribosomal protein 18* as an internal control. The primers used were as follows: *canine collagen*, *type1*, *alpha2* (*COL1A2*) primers: sense (5′-CCCAGCCAAGAACTGGTACAGAA-3′), antisense (5′-CGCATGAAGGCGAGTTGAGTAG-3′); *canine collagen*, *type3*, *alpha1* (*COL3*) primers: sense (5′-CATCTCGGCACAGCAGCAA-3′), antisense (5′-CAGATCCTGAGTCACAGACGCATA-3′); *canine tissue inhibitor of metalloproteinase 1* (*TIMP-1*) primers: sense (5′-TTCACCAAGACCTATGCTGCTGCTG-3′), antisense (5′-AGTTGCATATCCCTGGCTCTC-3′).

### Statistical analysis

Data were analyzed using a Student’s t-test and paired t-test. Values of p < 0.05 were considered statistically significant. Data are presented as the mean ± standard deviation.

## Results

### Assessment of fibrotic area

**Fig 3:**
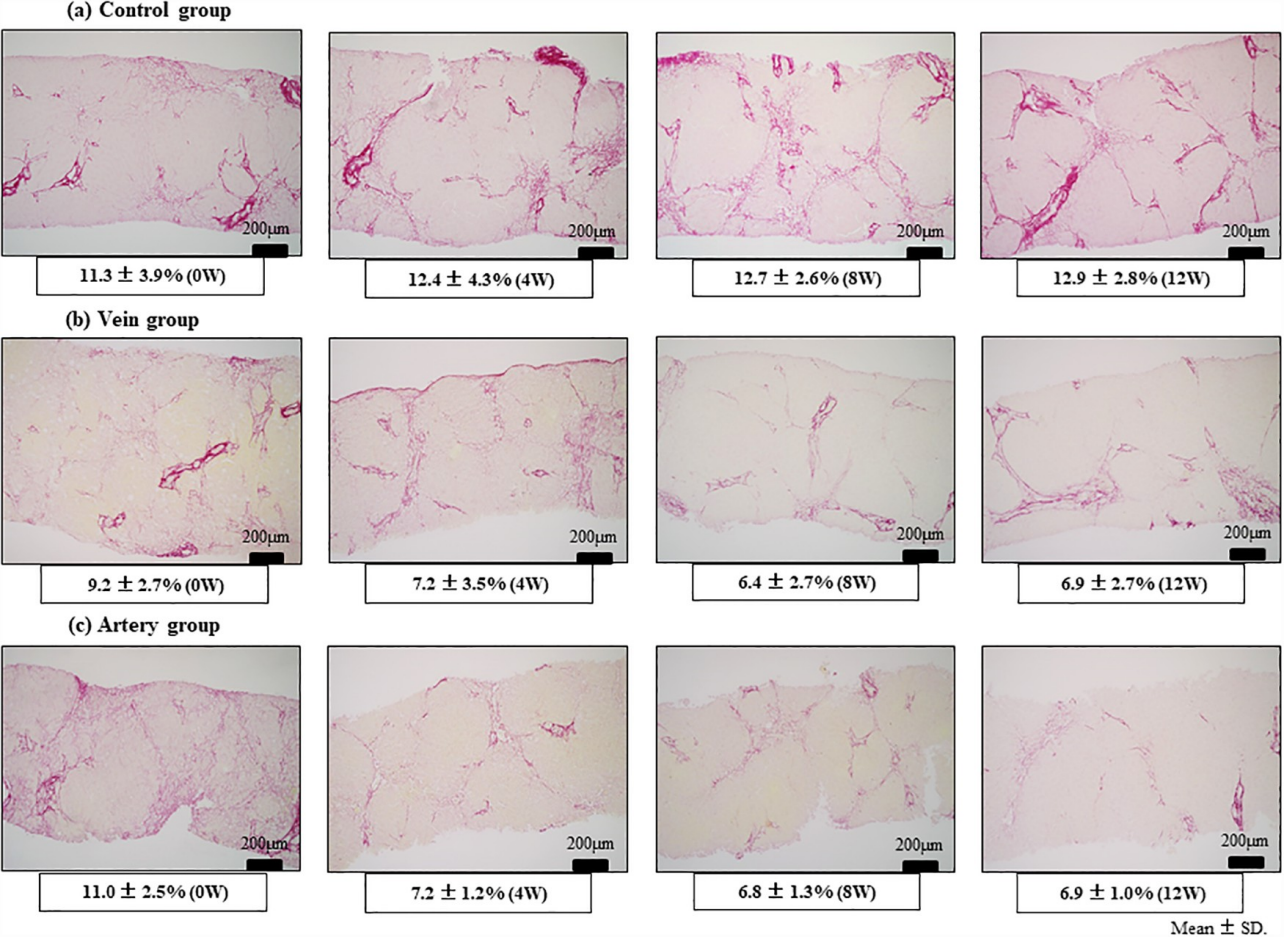
Liver fibrosis in different treatment groups as assessed by Sirius-red staining. The canines were administered repeated carbon tetrachloride (CCl_4_) injections to induce liver fibrosis and treated with bone marrow-derived mesenchymal stem cells (BMSCs) via the peripheral vein (Vein group) or hepatic artery (Artery group). (a) In the Control group, the fibrosis level increased over time from 11.3 ± 3.9% (0W) to 12.4 ± 4.3% (4W), 12.7 ± 2.6% (8W), and 12.9 ± 2.8% (12W). (b) In the Vein group, the fibrosis level decreased from 9.2 ± 2.7% (0W) to 7.2± 3.5% (4W), 6.4 ± 2.7% (8W), and 6.9 ± 2.7% (12W). (c) In the Artery group, the fibrosis level decreased significantly from 11.0 ± 2.5% (0W) to 7.2 ± 1.2% (4W; p < 0.05), 6.8 ± 1.3% (8W; p < 0.05), and 6.9 ± 1.0% (12W; p < 0.05). 4W, 8W, and 12W denote weeks post-BMSC administration.

**Fig 4:**
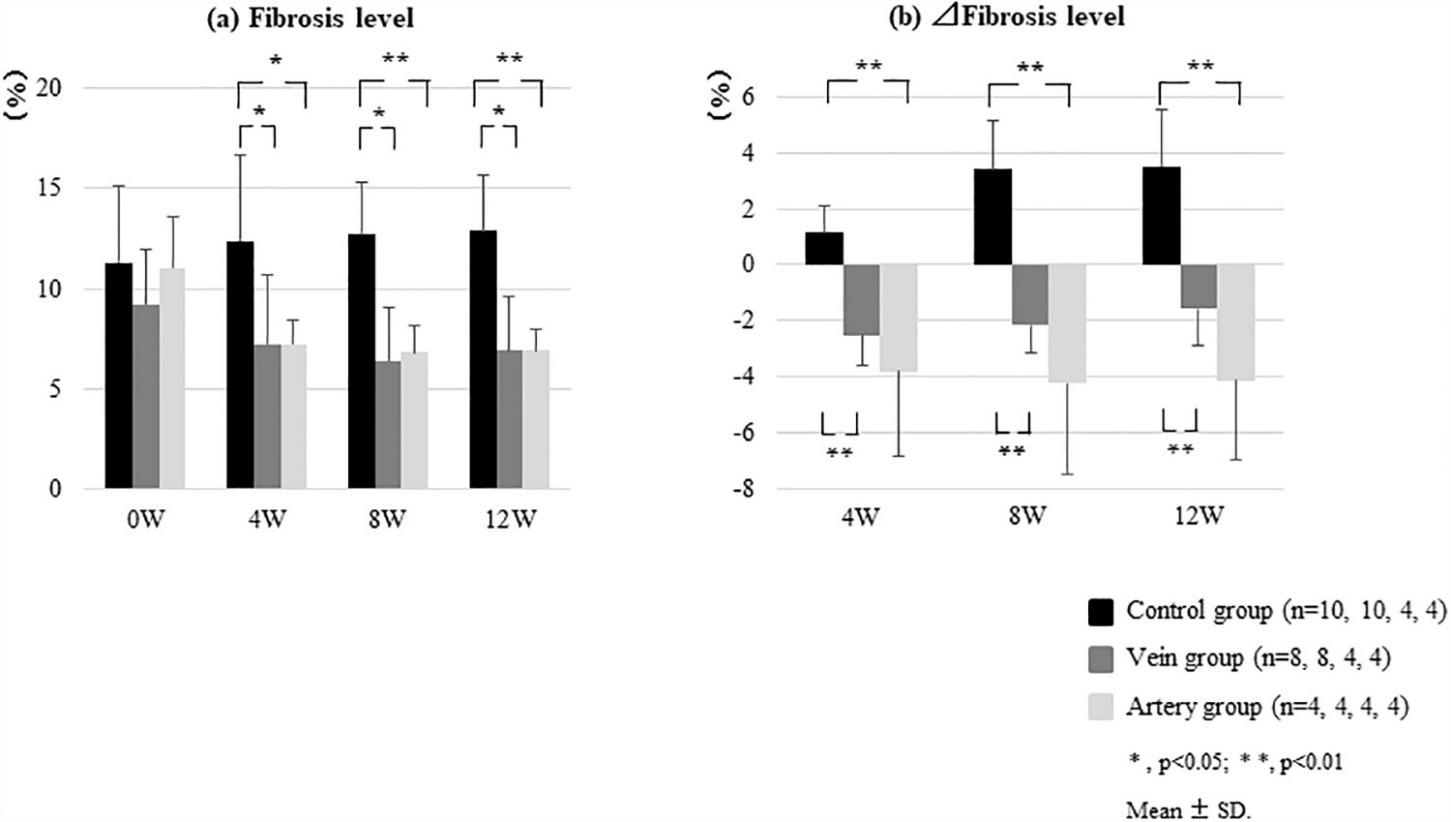
Quantification of liver fibrosis assessed by Sirius red staining in different treatment groups. The canines were administered repeated carbon tetrachloride (CCl_4_) injections to induce liver fibrosis and treated with bone marrow-derived mesenchymal stem cells (BMSCs) via the peripheral vein (Vein group) or hepatic artery (Artery group). (a) Significant improvements in fibrosis level were found in both the Vein and Artery groups with respect to that in the Control group at 4, 8, and 12 weeks post-BMSC administration. (b) With respect to the change in (Δ) fibrotic area, a significant improvement in fibrosis was found at 4 weeks post-BMSC administration in the Vein (Δfibrotic area: −2.5 ± 1.1%) and Artery groups (−3.8 ± 3.0%) relative to that in the Control group (+0.9 ± 1.0%). A greater effect was found in the Artery group, and this effect was maintained even after 8 (−4.3 ± 3.2%) and 12 (−4.2 ± 2.8%) weeks.

First, adverse events associated with catheter implantations were not observed, and US-guided liver biopsy was performed over time. Bridging fibrosis was confirmed at 0W based on Sirius red staining, which demonstrated pseudo-lobules in some samples. The fibrosis level in the Control group increased from 11.3 ± 3.9% (0W) to 12.4 ± 4.3% (4W), 12.7 ± 2.6% (8W), and 12.9 ± 2.8% (12W), whereas that in the Vein group decreased from 9.2 ± 2.9% (0W) to 7.2± 3.5% (4W), 6.4 ± 2.7% (8W), and 6.9 ± 2.7% (12W) (Fig 3a and 3b and S1 Table). In the Artery group, the fibrosis level also significantly decreased from 11.0 ± 2.5% (0W) to 7.2 ± 1.2% (4W; p < 0.05), 6.8 ± 1.3% (8W; p < 0.05), and 6.9 ± 1.0% (12W; p < 0.05; Figs 3C and 4A). In terms of the Δfibrosis area 4 weeks after BMSC infusion, there was a significant improvement in fibrosis in both the Vein (Δfibrosis area: −2.5 ± 1.1%) and Artery groups (−3.8 ± 3.0%) relative to that in the Control group (+0.9 ± 1.0%). However, the Artery group showed a greater effect, and this effect was maintained even at 8 (−4.3 ± 3.2%) and 12 (−4.2 ± 2.8%) weeks (Fig 4b).

### ICG test results

**Fig 5:**
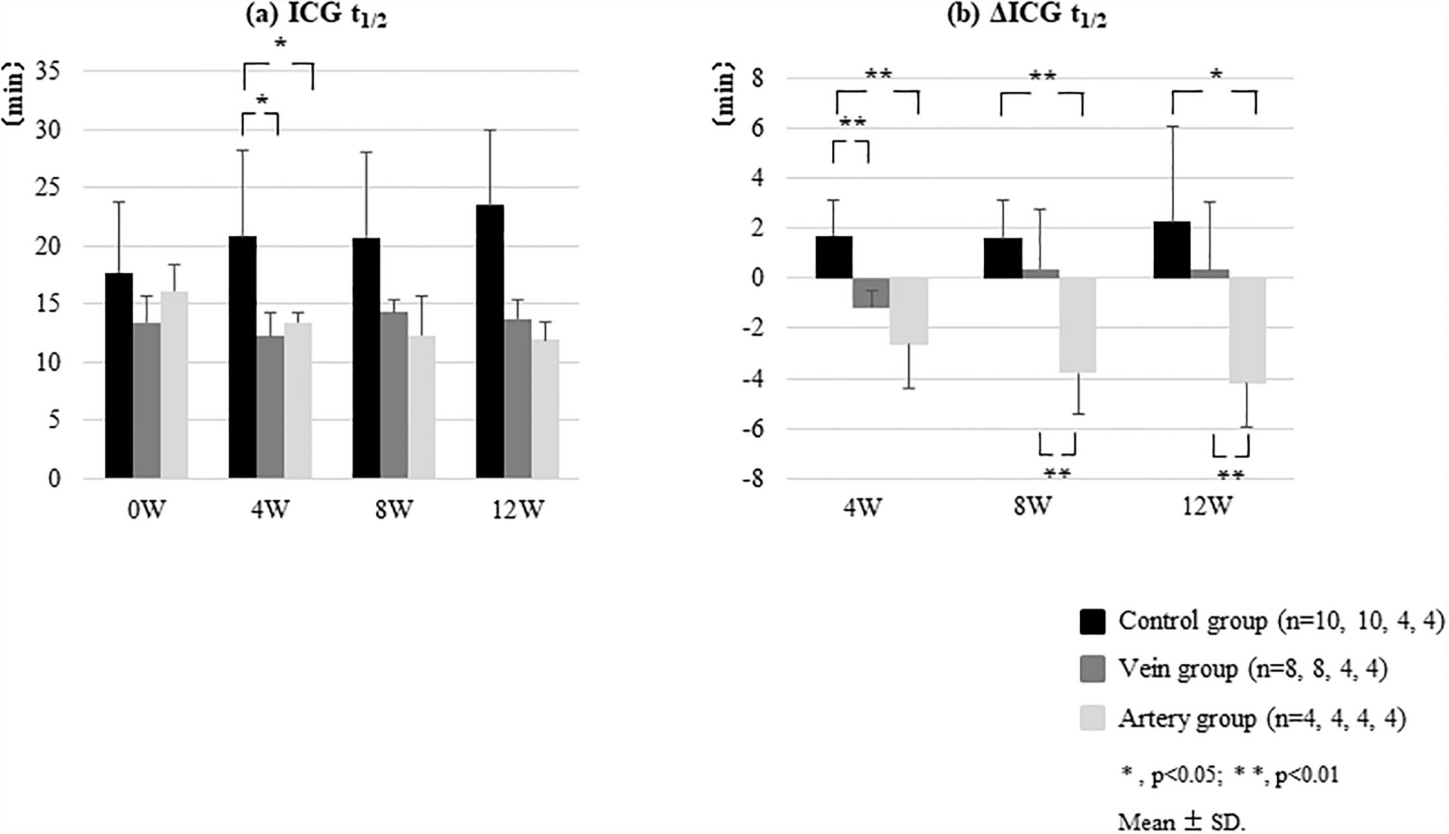
Therapeutic effects of transfused BMSCs in a CCl_4_-induced canine liver fibrosis model. After the induction of liver fibrosis, canines were treated with BMSCs via the peripheral vein (Vein group) or hepatic artery (Artery group). (a) Indocyanine green (ICG) half-life (t_1/2_) in the Vein group was slightly decreased at 4 weeks post-BMSC administration, from 13.4 ± 2.3 min (0W) to 12.2 ± 2.0 min (4W), but increased from 8 weeks onward to 14.3 ± 1.1 min (8W) and 13.7 ± 1.7 min (12W). In the Artery group, the ICG t_1/2_ decreased from 16.1 ± 2.3 min (0W) to 13.4 ± 0.8 min (4W), and this value was subsequently maintained (12.3 ± 3.3 min (8W), 12.0 ± 1.5 min (12W)). (b) The ΔICG t_1/2_ value was significantly decreased in the Vein group compared to that in the Control group at 4 weeks post-BMSC administration (ΔICG t_1/2_ (min): −1.2 ± 0.7 vs. 1.7 ± 1.4; p < 0.01). Moreover, the ICG t_1/2_ in the Artery group (n = 4) was significantly decreased at 8 weeks (Δt_1/2_: −4.2 ± 1.7 min vs. +0.4 ± 2.7 min; p < 0.01) and 12 weeks after BMSC administration (Δt_1/2_: −4.2 ± 1.7 min vs. +0.4 ± 2.7 min; p < 0.05) even when compared to that in the Vein group. All error bars represent the standard deviation of the mean.

The ICG t_1/2_ increased over time in the Control group from 17.7 ± 3.4 min (0W) to 20.8 ± 6.3 min (4W), 20.7 ± 4.1 min (8W), and 23.5 ± 5.0 min (12W). In the Vein group, the ICG t_1/2_ slightly decreased at 4 weeks, from 13.4 ± 2.3 min (0W) to 12.2 ± 2.0 min (4W); however, from 8 weeks on, it increased to 14.3 ± 1.1 min (8W) and 13.7 ± 1.7 min (12W). In the Artery group, the ICG t_1/2_ decreased from 16.1 ± 2.3 min (0W) to 13.4 ± 0.8 min (4W), and this value was subsequently maintained (12.3 ± 3.3 min (8W) and 12.0 ± 1.5 min (12W; Fig 5a and S2 Table).

The ΔICG t_1/2_ values in the Vein group (n = 8) were significantly lower than those in the Control group (n = 10) at 4 weeks following BMSC administration (ΔICG t_1/2_ (min): −1.2 ± 0.7 vs. 1.7 ± 1.4; p < 0.01); however, no obvious significant differences were found at 8 or 12 weeks post-BMSC administration. In the Artery group (n = 4), ICG t_1/2_ values were significantly lower at 8 weeks (Δt_1/2_: −4.2 ± 1.7 min vs. +0.4 ± 2.7 min; p < 0.01) and 12 weeks after BMSC administration (Δt_1/2_: −4.2 ± 1.7 min vs. +0.4 ± 2.7 min; p < 0.05) even when compared to those in the Vein group (Fig 5b).

### Biochemical results

In the Control group, albumin and AT3 gradually tended to become exacerbated. In the Vein group, although improvement was observed at 4 weeks, subsequent gradual exacerbation was observed. However, in the Artery group, this improvement at 4 and 8 weeks was maintained at 12 weeks (Table 1 and S3 Table).

**Table 1:** Clinical laboratory test results.

	Control group				Vein group				Artery group																																																					
	0W (n = 10)	4W (n = 10)	8W (n = 4)	12W (n = 4)	0W (n = 8)	4W (n = 8)	8W (n = 4)	12W (n = 4)	0W (n = 4)	4W (n = 4)	8W (n = 4)	12W (n = 4)
Alb (g/dL)	2.5±0.3	2.3±0.3	2.4±0.2	2.4±0.3	2.7±0.2	2.8±0.2	2.7±0.2	2.5±0.1	2.5±0.1	2.6±0.1	2.5±0.1	2.6±0.1
AST (U/L)	60.2±31.5	85.1±39.9	81.7±83.8	70.3±39.9	59.5±36.3	50.1±21.9	60.1±31.5	42.3±14.5	83.0±53.8	54.0±20.6	51.5±28.3	35.0±5.6
ALT (U/L)	662.1±663.4	850.2±498.8	619.0±717.2	506.8±232.3	374.8±520.1	327.6±382.4	472.8±572.5	232.5±274.9	689.5±728.8	649.8±583.1	506.8±615.5	221.5±144.8
LDH (U/L)	163.3±100.2	169.4±66.1	96.8±23.4	89.5±24.5	146.5±21.1	153.8±73.6	168.5±75.8	174.8±79.9	146.0±41.8	117.8±26.3	130.0±46.2	118.8±45.3
PT (sec)	9.5±1.8	8.9±1.2	9.3±2.4	9.7±2.5	7.4±0.7	7.3±0.9	7.1±0.8	6.7±0.5	9.7±3.1	8.3±1.0	8.3±1.0	8.0±1.0
AT3 (%)	102.3±21.0	97.9±14.8	97.3±25.8	94.8±30.4	112.1±11.7	116.5±11.4	113.3±17.7	108.0±9.9	99.0±15.3	106.5±12.9	108.3±9.1	113.8±7.8

Alb, albumin; AST, aspartate aminotransferase; ALT, alanine aminotransferase; LDH, lactate dehydrogenase; PT, prothrombin time; AT3, antithrombin 3. Data are expressed as mean ± standard deviation.

### Expression of liver fibrosis-related genes

**Fig 6:**
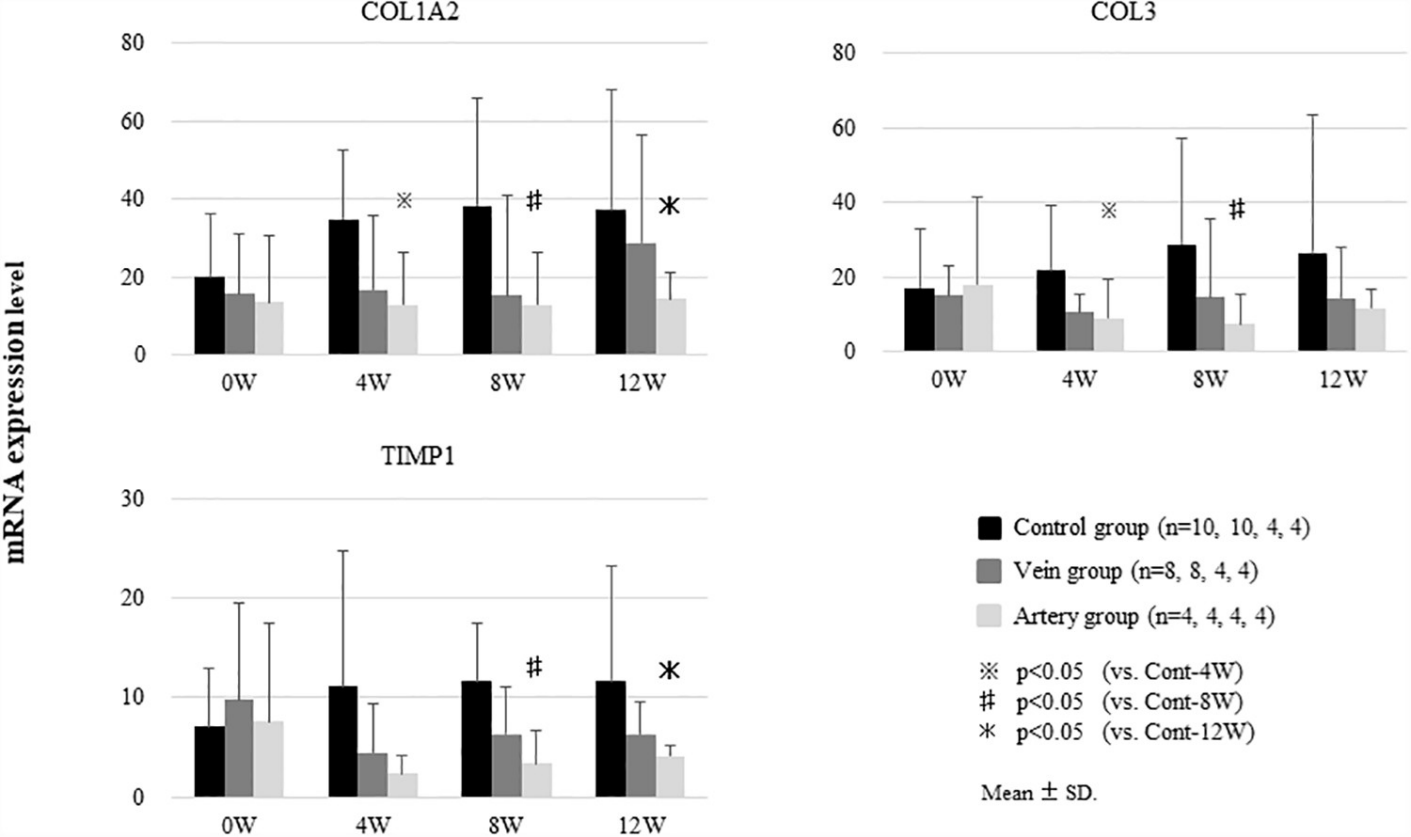
Expression of liver fibrosis-related genes after the induction of liver fibrosis and BMSC treatment. After the induction of liver fibrosis, canines were treated with BMSCs via the peripheral vein (Vein group) or hepatic artery (Artery group). The expression of *COL1A2*, *COL3*, and *TIMP-1* mRNA tended to decrease in the Vein group compared to that the Control group, although the differences were not significant. In the Artery group, the expression levels of *COL1A2*, *COL3*, and *TIMP-1* were significantly lower than those in the Control group at 4, 8, and 12 weeks. Data show the mean ± standard deviation.

The expression of *COL1A2*, *COL3*, and *TIMP-1* mRNA, which encode proteins involved in hepatic fibrosis, was analyzed. Expression levels of all genes tended to decrease in the Vein group compared to those in the Control group, although the differences were not significant. However, in the Artery group, the levels of all genes were significantly decreased compared to those in the Control group at 4, 8, and 12 weeks (Fig 6).

### Safety evaluations

**Fig 7:**
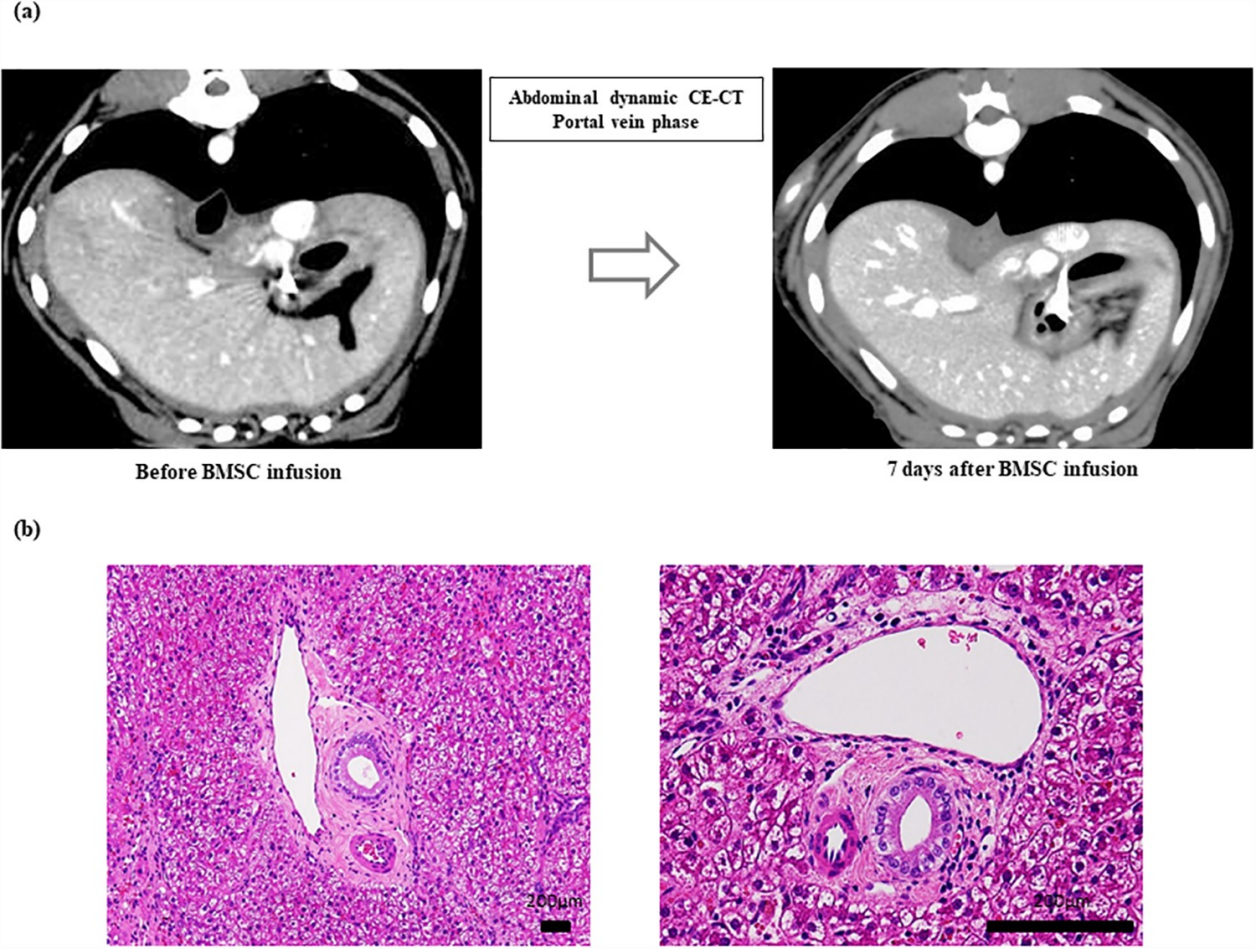
Contrast-enhanced computed tomography (CE-CT) and photomicrographs of liver tissue samples after BMSC application. (a) After the infusion of CE-CT, no evidence of liver infarction and the absence of major portosystemic shunts (PSS) were observed. (b) Arterial embolization and biliary necrotic images were not observed in the liver tissues following hematoxylin and eosin staining.

Post-infusion contrast-enhanced CT showed no sign of liver infarction and the absence of major PSS (Fig 7a). Further, we did not observe oxygen desaturation, a remarkable change in pulse rate, or a decline in general condition after BMSC infusion in any of the canines including the canine used to test an increased dose. Moreover, in the canine infused with a higher number of BMSCs (1.2 × 10^6^ cells/kg), blood tests showed no elevation in either serum lactate dehydrogenase concentrations or hypercoagulability (Table 2). Moreover, arterial embolization and necrotic biliary areas were not observed based on hematoxylin and eosin staining of the liver tissue (Fig 7b).

**Table 2:** Clinical laboratory test results in the safety evaluation.

	Before	Day 1	Day 3	Day 5	Day 7
Alb (g/dL)	2.6±0.2	2.9±0.3	3.1±0.2	3.1±0.3	2.8±0.2
AST (U/L)	37.2±8.5	47.1±9.3	33.5±5.9	36.1±12.4	33.8±9.3
ALT (U/L)	1302.1±532.3	1166.5±368.9	799.1±132.4	575.8±122.9	387.3±92.3
LDH (U/L)	41.6±14.5	203.1±86.1	99.2±22.8	64.0±19.2	70.7±27.9
PT (sec)	7.5±1.2	7.0±1.1	6.8±0.9	6.6±1.1	6.8±0.8
FDP (μg/mL)	1.0±0.1	1.4±0.1	2.4±0.2	2.4±0.2	1.4±0.1
D-dimer (μg/mL)	0.4±0.1	0.5±0.1	1.2±0.1	1.1±0.1	0.5±0.1
AT3 (%)	99.2±12.5	126.3±21.8	150.2±26.1	149.9±19.2	132.7±24.5

Alb, albumin; AST, aspartate aminotransferase; ALT, alanine aminotransferase; LDH, lactate dehydrogenase; PT, prothrombin time; FDP, fibrin degradation products; AT3, antithrombin 3. Data are expressed as mean ± standard deviation.

## Discussion

The present study showed the efficacy and safety of hepatic artery infusion using cultured autologous BMSCs based on a canine liver fibrosis model.

In a previous study, we placed a port and a catheter in beagles, and using this system, administered CCl_4_ over a 10-week period to induce liver fibrosis. We then administered cultured autologous BMSCs via peripheral veins and confirmed the safety and efficacy of this approach for the treatment of fibrosis [11].

In an attempt to increase the efficacy of this treatment, we considered the selective administration of BMSCs into the liver via the hepatic artery to facilitate more effective liver regeneration. Hence, in this study, we administered cultured autologous BMSCs into the hepatic artery, and compared treatment efficacy and safety between the two administration routes.

Using canines, we established a technique based on abdominal angiography for the administration of BMSCs via the hepatic artery, which is used routinely in medical practice. Canine abdominal blood vessels differ from those of humans in that after an artery branches from the common hepatic artery toward the outer right liver lobe, the gastroduodenal artery branches into the artery leading to the inner right lobe and the artery leading to the left lobe. In a joint study carried out at the Veterinary Surgery Laboratory of Yamaguchi University Joint Faculty of Veterinary Medicine, the abdominal vascular course of 30 beagles was studied using 3-D CT, and in 80% of these animals, the blood vessel to the outer right lobe branched before the gastroduodenal artery [12]. Based on this vascular anatomy, which is different from that of humans, we introduced a catheter into the left hepatic artery, the right medial lobe branch, and the right lateral lobe branch, and administered BMSCs at 2 × 10^5^ cells/kg 1 × 10^5^ cells/kg, and 1 × 10^5^ cells/kg, respectively.

We also assessed the ICG t_1/2_ to evaluate liver function. Boothe et al. proved the diagnostic benefits of using ICG disposition kinetics as a method to evaluate hepatic function in canines with progressive liver disease [13]. However, in cases with PSS, the results of ICG testing might exaggerate the diminished functional reserve [14, 15]. Since we confirmed the absence of PSS in our experimental model by CE-CT (Fig 7A), the prolonged ICG t_1/2_ observed in our canine model is a true reflection of liver function.

At 4 weeks after the administration of BMSCs, the ICG t_1/2_ increased in the Control group but decreased in the Vein and Artery groups. Moreover, whereas the ICG t_1/2_ increased in the Vein group at 8 weeks post-BMSC administration, the effects of treatment were maintained in the Artery group at 8 and 12 weeks post-administration. Consequently, the effects of BMSCs injected via a peripheral vein in this canine liver fibrosis model were suggested to last for approximately 4 weeks. In contrast, with the administration of BMSCs via the hepatic artery, it is anticipated that these effects can be maintained for at least 12 weeks after administration.

Boothe et al. induced hepatic disease in canines with dimethylnitrosamine, and reported that Alb, T-bil, and prothrombin time were only slightly different between mild and severe hepatic disease groups, and that AT3 activity correlated with hepatic disease severity, similar to that observed in ICG experiments [13]. In our study, AT3 decreased in the Vein group 8 weeks after BMSC administration, similar to results obtained for the ICG experiment, although the decrease in the Artery group was maintained at 8 and 12 weeks post-administration.

Whereas the expression of mRNA involved in hepatic fibrosis tended to be lower in the Vein group than the Control group, it was significantly lower at 4, 8, and 12 weeks in the Artery group, compared to that in the Control group. Kanemoto et al. reported that the expression of these genes correlates well with the histologic degree of fibrosis in canines [16]. Hence, assuming that the reduced expression of these genes in the Artery group is consistent with the degree of fibrosis (as suggested by the results of Kanemoto et al.), it appears that a stronger anti-fibrotic effect occurred in the Artery group than in the Vein group.

The mechanisms underlying BMSC-mediated improvements in fibrosis remain to be clarified. Several studies have suggested that mesenchymal stem cells (MSCs) exert their effects on different aspects of fibrosis to facilitate this improvement. The first of these is the effect of MSCs on reducing liver cell damage. MSCs are believed to prevent liver cell damage by suppressing oxidative stress induced at the time of injury and by downregulating inflammatory cytokines such as TNF-α and IFN-γ [17, 18]. Therefore, they are thought to have an anti-inflammatory effect. At the same time, some reports have suggested that MSCs have immunosuppressive and immunoregulatory effects, and that they inhibit T cell proliferation during liver inflammation and promote regulatory T cell proliferation [19, 20]. It has also been reported that MSCs tend to suppress inflammation through their effects on macrophages and dendritic cells, which indirectly inhibits the action of stellate cells and is involved in the inhibition of fibrosis [21]. In addition, there are many aspects related to the direct effects of MSCs that are unclear, although numerous reports have reported improved fibrosis following the administration of MSCs. Thus, based on these effects on the abatement of liver disease and the suppression of inflammation, it appears likely that MSCs exert a secondary effect by indirectly inducing endogenous mechanisms to improve fibrosis. Further clarification of such mechanisms will be an area for future work.

The optimal route of MSC delivery to the liver is an important concern. Most studies have used an intravenous route, although a large proportion of MSCs that are injected via this route become trapped in the lungs upon first passage [22–25]. Moreover, several studies have revealed that intra-arterial injection can significantly decrease the number of MSCs that are trapped within the lungs, thus increasing their uptake by other organs, especially the liver [26–28]. We, therefore hypothesized that the administration of MSCs via the hepatic artery, rather than via a peripheral vein, would prevent MSCs from becoming trapped in the lungs, ensuring greater accumulation in the liver, and leading to improved liver function and the suppression of liver fibrosis. However, even in our canine liver fibrosis model, the engraftment of administered cells needs to be analyzed and studied in the future. Suk et al. also reported that BMSC administration into the hepatic artery could augment improvements in fibrosis and Child-Pugh scores, an index of liver function, in the group administered cells via both routes compared to those in the single administration group [29].

BMSCs can be cryopreserved once they have been cultured, allowing for frequent administration while reducing burden to the patient; this will likely lead to a more lasting therapeutic effect. Since multiple administrations of cryopreserved autologous BMSCs will likely bring about greater therapeutic efficacy for hepatic fibrosis, it will be important to perform comparative studies to test this in the future.

In conclusion, we confirmed the efficacy and safety of the hepatic artery infusion of cultured autologous BMSCs for the treatment of liver fibrosis using a canine model, therefore providing non-clinical proof-of-concept for this approach.

## Supporting information

S1 Table Liver fibrosis assessed by Sirius red staining after the hepatic arterial infusion of cultured autologous bone marrow-derived mesenchymal stem cells. (Upper table) Fibrosis level (%) is the mean of the levels of Sirius red stained area in liver biopsied section. (Lower table) ΔFibrosis level (%) was calculated using the formula; Δfibrosis level (%) = fibrosis level (%) at 4W, 8W, or 12W − fibrosis level (%) at 0W. Control group (n = 10, 10, 4, 4), Vein group (n = 8, 8, 4, 4), Artery group (n = 4, 4, 4, 4). W, week; SD, the standard deviation of the mean.
(XLSX)

S2 Table Indocyanine green (ICG) half-life (t_1/2_) after the hepatic arterial infusion of cultured autologous bone marrow-derived mesenchymal stem cells. (Upper table) the mean and SD of ICG t_1/2_ (min). (Lower table) the mean and SD of ΔICG t_1/2_ (min)_._ ΔICG t_1/2_ (min) was calculated using the formula; ΔICG t_1/2_ (min) = ICG t_1/2_ (min) at 4W, 8W, or 12W –ICG t_1/2_ (min) at 0W. Control group (n = 10, 10, 4, 4), Vein group (n = 8, 8, 4, 4), Artery group (n = 4, 4, 4, 4). W, week; SD, the standard deviation of the mean.
(XLSX)

S3 Table Clinical laboratory test after the hepatic arterial infusion of cultured autologous bone marrow-derived mesenchymal stem cells. Alb, albumin; AST, aspartate aminotransferase; ALT, alanine aminotransferase; LDH, lactate dehydrogenase; PT, prothrombin time; AT3, antithrombin 3. Data are expressed as mean ± standard deviation (SD). Control group (n = 10, 10, 4, 4), Vein group (n = 8, 8, 4, 4), Artery group (n = 4, 4, 4, 4). W, week; C-1~C-10, each canine in Control group; V-1~V-8, each canine in Vein group, A-1~A-4, each canine in Artery group.
(XLSX)
